# Detection of Reverse Transcriptase LAMP-Amplified Nucleic Acid from Oropharyngeal Viral Swab Samples Using Biotinylated DNA Probes through a Lateral Flow Assay

**DOI:** 10.3390/bios13110988

**Published:** 2023-11-17

**Authors:** Saloni Agarwal, Mojdeh Hamidizadeh, Frank F. Bier

**Affiliations:** 1 Institute for Biochemistry and Biology, Chair of Molecular Bioanalysis and Bioelectronics, University of Potsdam, Karl-Liebknecht-Strasse 24/25, 14476 Potsdam, Germany; agarwal@uni-potsdam.de (S.A.); mojdeh.hamidizadeh@uni-potsdam.de (M.H.); 2 Institute for Molecular Diagnostics and Bioanalysis-IMDB gGmbH, Am Mühlenberg 10, 14476 Potsdam, Germany

**Keywords:** RT-LAMP, LFA, NAAT-LFA, semi-quantitative, surveillance-based diagnostics

## Abstract

This study focuses on three key aspects: (a) crude throat swab samples in a viral transport medium (VTM) as templates for RT-LAMP reactions; (b) a biotinylated DNA probe with enhanced specificity for LFA readouts; and (c) a digital semi-quantification of LFA readouts. Throat swab samples from SARS-CoV-2 positive and negative patients were used in their crude (no cleaning or pre-treatment) forms for the RT-LAMP reaction. The samples were heat-inactivated but not treated for any kind of nucleic acid extraction or purification. The RT-LAMP (20 min processing time) product was read out by an LFA approach using two labels: FITC and biotin. FITC was enzymatically incorporated into the RT-LAMP amplicon with the LF-LAMP primer, and biotin was introduced using biotinylated DNA probes, specifically for the amplicon region after RT-LAMP amplification. This assay setup with biotinylated DNA probe-based LFA readouts of the RT-LAMP amplicon was 98.11% sensitive and 96.15% specific. The LFA result was further analysed by a smartphone-based IVD device, wherein the T-line intensity was recorded. The LFA T-line intensity was then correlated with the qRT-PCR Ct value of the positive swab samples. A digital semi-quantification of RT-LAMP-LFA was reported with a correlation coefficient of R^2^ = 0.702. The overall RT-LAMP-LFA assay time was recorded to be 35 min with a LoD of three RNA copies/µL (Ct-33). With these three advancements, the nucleic acid testing-point of care technique (NAT-POCT) is exemplified as a versatile biosensor platform with great potential and applicability for the detection of pathogens without the need for sample storage, transportation, or pre-processing.

## 1. Introduction

Due to the COVID-19 pandemic, the definition and perspective of “diagnosis of an infection” have altered greatly [1]. During this time, it became imperative to apprehend the abilities and pitfalls of currently globally employed modern diagnostic procedures, primarily in low-resource nations [2]. Though the number of cases reported by healthcare centres worldwide was considerably large, the probable positive number of cases (including the asymptomatic cases) was even higher and unreported due to a lack of surveillance-based diagnostics [3]. One of the main reasons for this unbalanced tally was the global practice of using time-consuming diagnostic methods with expensive equipment: quantitative real-time PCR (qRT-PCR) or real-time PCR (RT-PCR) [4,5]. qRT-PCR, being the gold-standard method, caused delays in delivering diagnostic results, therefore, these methods do not include surveillance of asymptomatic infections [6]. Consequently, the highlighted disadvantages of PCR during this period aided in directing fundamental and applied research towards new-generation, rapid, and on-site/at-home-based point-of-care (POC) diagnostic techniques.

During the past three years, the improvements and advancements in POC diagnostics research have highlighted the versatility, efficiency, and efficacy of isothermal amplification techniques, especially loop-mediated isothermal amplification (LAMP). Methods like LAMP [7,8] or reverse transcriptase LAMP (RT-LAMP) [9], recombinase polymerase amplification (RPA) [10,11], and helicase-dependent amplification (HDA) or reverse transcriptase HDA (RT-HDA) [12] have emerged as potential alternatives to RT-PCR. More recently, modified isothermal techniques, like SHARP [13] and CRISPR-based detection methods (SHERLOCK, DETECTR, etc.) [14,15,16], have also demonstrated a similar catalogue of PCR applications. Out of all these isothermal techniques, RT-LAMP has been most extensively compared to RT-PCR using parameters such as sample pre-treatment (RT-LAMP is more flexible to biological and chemical inhibitors than RT-PCR), analytical time and robustness (RT-LAMP amplification takes less than 30 min, unlike RT-PCR, which takes >3 h), and assay efficacy (RT-LAMP nears the sensitivity of RT-PCR, which is 68–80% sensitive and 90–95% specific, according to WHO guidelines). These characteristics make RT-LAMP equivalent to RT-PCR and pave the way for developing RT-LAMP-based POC technological biosensor platforms [8,17,18].

Incorporating modifications or labels into the LAMP/RT-LAMP amplification product (amplicon) during the amplification process of LAMP and RPA has also been successfully demonstrated. Introducing biotin and FITC/FAM labels [19] enables the readout on a paper-based, sensitive immunochromatographic lateral flow assay (LFA) platform with high efficacy and synergism [20,21,22].

Oro-nasopharyngeal swab samples (widely collected for COVID-19 sampling) for viral infections are generally transported and stored in a universal viral transport medium (uVTM). uVTMs are buffer solutions consisting of sugars, salts, and pH indicators that preserve the collected viral sample but inhibit virus multiplication. Such a complex matrix usually requires pre-treatment to extract nucleic acid for conventional diagnostic procedures, as uVTM ingredients are inhibitory to most DNA polymerases available for nucleic acid amplification. In a usual diagnostic procedure, sample pre-treatment increases delays before the actual diagnostic assay can be performed and analysed [19]. Each molecular diagnostic technique uses extracted and purified RNA or DNA templates [8,20,23]. Hence, the complex uVTM composition and the human oro-nasopharyngeal swab sample have not yet been used directly in diagnostic assays. Alternative methods have been explored to extract or filter viral RNA, e.g., through a Chelex resin-based filtration of RNA during viral testing [24] or using a NaOH lysis buffer after collecting the swab sample [22]; however, the swab sample in uVTMs “as is” has not yet been examined.

Individual laboratories and institutes have their own qPCR standards, from which the range of the viral load concentration in the sample can be determined. The result obtained from qRT-PCR diagnostics is given as a Ct value, i.e., the cycle threshold where the cDNA sample begins exponential amplification. This Ct value corresponds to a certain viral load concentration in the sample but is not yet the absolute quantification [25]. However, other researchers have found that the viral load concentration in a sample is inversely proportional to the Ct value from qRT-PCR. Alternatively, immunochromatographic LFA-based readout platforms showcase great potential to produce similar semi-quantitative results [20,26]. The LFA produces a test line of a certain intensity, as does the control line (intrinsic chromatographic control for the LFA). Test line intensity can be correlated with the Ct value from the qRT-PCR and, hence, semi-quantified using an IVD device or smartphone [27].

**Figure 1:**
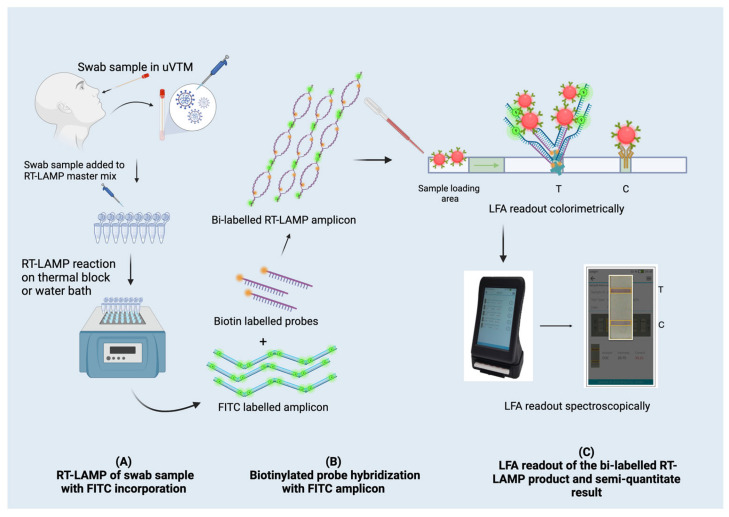
Concept-wise diagrammatic representation of the POCT setup of RT-LAMP-LFA to detect viral RNA in swab samples. (**A**) RT-LAMP of swab samples with FITC incorporation is the first concept of enzymatically incorporating FITC into the RT-LAMP amplicon. (**B**) Biotinylated probe hybridization with FITC amplicon is the second concept to produce a bi-labelled RT-LAMP amplicon. (**C**) LFA readout of the bi-labelled RT-LAMP product and semi-quantitative results is the third concept where a T-line and C-line are produced on the LFA and then the result is readout by an IVD device for semi-quantification purposes.

In this work, we emphasise the results for three improved aspects (see Figure 1): Firstly, we show that nucleic acid pre-preparation or extraction could be eliminated by diluting the primary sample in the uVTM itself. Secondly, we demonstrate the biotinylated DNA probes directed to the amplicon region, which facilitate the easy adaptation of the RT-LAMP product to the LFA and increase the overall efficiency of the two combined assays. Thirdly, a smartphone-based digitally supported readout of the LFA illustrates that the LFA-test band intensities correlate with viral load as measured using Ct values from qRT-PCR. With these three improvements, the nucleic acid testing-point of care technique (NAT-POCT) exemplifies a versatile biosensor platform with great potential and applicability.

## 2. Materials and Methods

### 2.1. SARS-CoV-2 Swab Sample

The positive and negative swab samples were collected and stored in a universal viral transport medium (uVTM) by In.vent Diagnostica GmbH (Henningsdorf, Germany). The received swab samples were heat inactivated at 56 °C for 30 min [28]. The uVTM consists of NaCl (0.8%), KCl (0.04%), CaCl_2_ (0.014%), MgSO_4_.7H_2_O (0.02%), Na_2_HPO_4_.7H_2_O (0.012%), KH_2_PO_4_ (0.006%), NaHCO_3_ (0.035%), C_6_H_12_O_6_ (0.1%), and phenol red sodium salt (0.002%) as ingredients. The positive samples were screened using the RT-PCR method for ORF1a and *E*-gene in the CT range of 18 to 32 using an ampliCube Coronavirus SARS-CoV-2 test kit from Mikrogen© (Neuried, Germany).

### 2.2. Reverse Transcriptase LAMP

The RT-LAMP primer set for the *N*-gene of SARS-CoV-2 was designed using the primer explorer software V5 at Fraunhofer-IZI-BB, Golm, Germany (see Table A1). The primers were purchased from Biomers.net GmbH (Ulm, Germany).

The RT-LAMP reaction mixture was adapted from Agarwal et al. [27], with the following enzyme cocktail: (1) *Bst* 3.0 polymerase (working horse enzyme), (2) helicase (to reduce the primer–dimer background), and (3) reverse transcriptase (enhance RTase activity and boost rapidity of RT-LAMP). The RT-LAMP reaction mixture was prepared according to Table A2 for a 25 µL reaction volume.

### 2.3. RT-LAMP Programme

The LAMP programme was run in a ProfessionalTRIO thermocycler from Biometra (Analytik Jena GmbH, Germany). The RT-LAMP programme was adapted from Agarwal et al. [27] and applied as mentioned in Table A3.

### 2.4. N-Gene-Specific Probe Design

**Figure 2:**
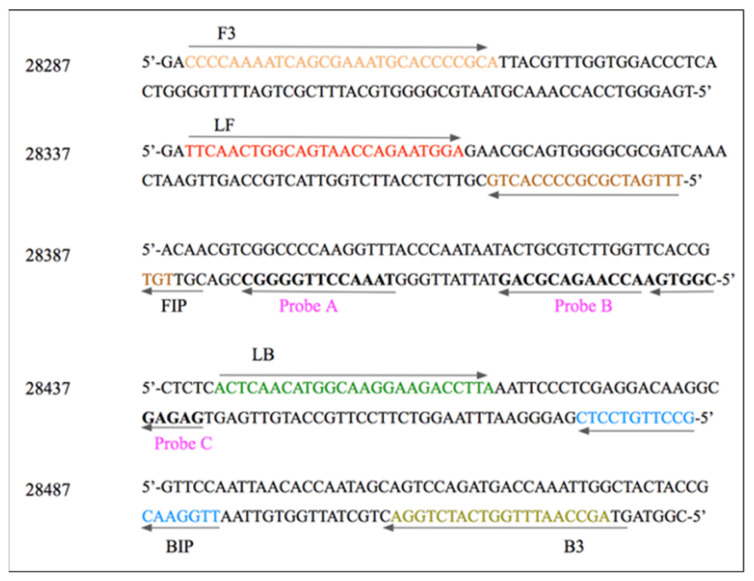
LAMP primer design and probe design for the *N*-gene (466 bp) of SARS-CoV-2. Six LAMP primers are designed to amplify 200 bp of the gene. The six primers are: forward outer loop primer (F3), backward outer loop primer (B3), loop-forming forward primer (LF), loop-forming backward primer (LB), forward inner primer (FIP), and backward inner primer (BIP). The three probes were devised in such a manner that there is no overlap with the primer sequences, and the probes could be uniquely determined. The probe design enabled biotin incorporation into the LAMP amplicon and eventually produced a bi-labelled LAMP product.

**Figure A1:**
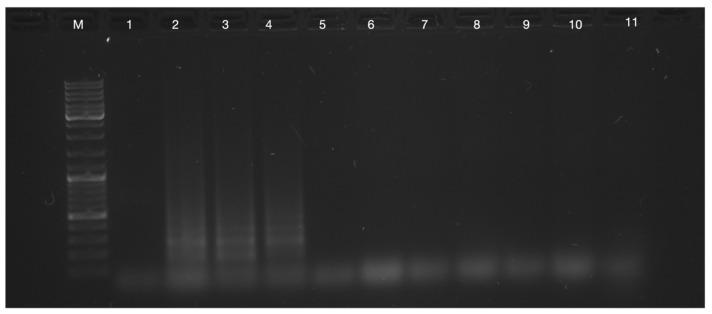
Temperature control for RT-LAMP amplification. The temperature range for RT-LAMP (20 min) investigated was from 47 °C to 65 °C with a gradient of 2 °C. In the gel image, M- 100 bp DNA marker, lane 1—NTC at 65 °C, lane 2—positive sample at 65 °C, lane 3—positive sample at 63 °C, lane 4—positive sample at 61 °C, lane 5—positive sample at 59 °C, lane 6—positive sample at 57 °C, lane 7—positive sample at 55 °C, lane 8—positive sample at 53 °C, lane 9—positive sample at 51 °C, lane 10—positive sample at 49 °C, lane 11—positive sample at 47 °C, respectively.

The *N*-gene of SARS-CoV-2 (gene-bank accession ID:MN908947.3) is a 466 bp nucleotide target sequence. The designed LAMP primers amplify a 200 bp region of the 466 bp sequence. A central region of 52 bp (out of the 200 bp) was targeted for designing three specific and unique DNA probes (see Figure 2). These probes had a Tm lower than the annealing Tm of the primers, facilitating specific hybridization to the amplicon after amplification. While the primers have an annealing temperature range of 60 °C to 65 °C (see Figure A1), the chosen hybridization temperatures for the probes were optimised at 52 °C to maintain the specificity of the POCT application. The range of hybridization temperatures tested was from 80 °C to 52 °C, and in all the cases, the hybridization result was the same.

### 2.5. Biotin-Labelled Probe Hybridization with the RT-LAMP Amplicon

The three specific DNA probes designed for *N*-gene detection were biotinylated at the 5′ end (see Table A4). After the RT-LAMP-FITC-labelled amplicon was produced, it was denatured at 52 °C for 2 min. Following denaturation, all three biotinylated probes (0.25 µM end concentration each) were added to the amplicon and made to hybridize for 2 min, followed by cooling at room temperature (see Table A5).

### 2.6. Lateral Flow Assay

**Figure 3:**
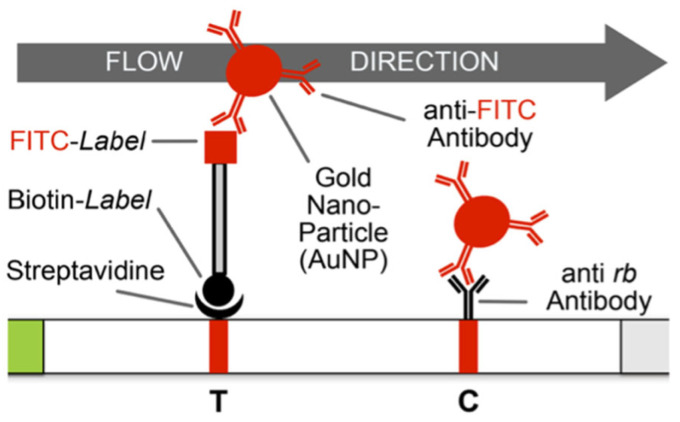
The principle for LFA readouts for positive and negative results is depicted in this schematic. For a negative LFA readout, only the control line (C) appears, whereas for a positive LFA readout, both the test (T) and control (C) lines appear. The bi-labelled LAMP amplicon mixed with the LFA running buffer is loaded onto the sample loading area on the test strip. The anti-FITC-Ab-coated gold nanoparticles (AuNPs) bind with the LAMP amplicon, and the complex migrates up the test strip by capillary force. The test line (T) appears on the test strip only when the amplicon has biotin incorporated in it and is captured by streptavidin, whereas the control line (C) appears as an intrinsic control for the validity of the test strip, where anti-rabbit-Ab captures the unbound AuNP-Anti-FITC-Ab particles. The absorption pad captures the leftover buffer and material. It takes about 3 min to read out the results (adapted from [29]).

The HybriDetect LFA kit was purchased from Milenia Biotec GmbH (Geißen, Germany) [29]. The test strip contains three main regions: (a) the sample loading area (containing AuNP-Anti-FITC/FAM antibody), (b) the conjugation pad, including the test (streptavidin) and control (anti-rabbit antibody) bands, and (c) an absorption pad (see Figure 3). The LFA has a characteristic principle for the sensitive and specific detection of LAMP products. The LAMP product needs to be labelled with biotin and FITC to be captured by streptavidin (on the test band) and AuNP-anti-FITC-Ab, respectively. In this study, we used the loop-forming (LF) primer labelled with FITC at the 5’ end (see Table A1) and the DNA probes labelled with biotin at the 5´ end (see Appendix A Table A4).

### 2.7. Agarose Gel Electrophoresis

The RT-LAMP amplicon was run in a 1.5% agarose gel to confirm the concatemeric “ladder-like” pattern of the LAMP product in 1X TAE buffer (Invitrogen, Waltham, MA, USA) at 95 V for 35 min. The agarose gel was visualized with a UV-Visualizer, E-box (Vilber, France).

### 2.8. Smartphone-Based In Vitro Diagnostic Device-Based LFA Readout

**Figure 4:**
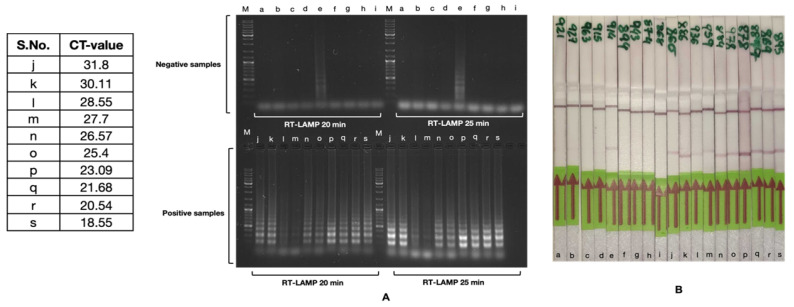
The incorporated table in the figure shows the Ct values of the 10 positive swab samples (j–s) corresponding to the gel image (**A**). The optimisation of RT-LAMP-LFA annealing time for 20 and 25 min with 10 negative and 10 positive SARS-CoV-2 swab samples was performed. (**A**) The gel shows 10 negative samples (upper half) subjected to RT-LAMP for 20 min and 25 min of annealing time. One of the negative samples (e) shows amplification, which could be a false positive from the RT-LAMP reaction or handling issues during reaction preparation. Ten positive samples (lower half) were also subjected to RT-LAMP for 20 min and 25 min of annealing time. A characteristic concatemeric pattern was observed for the RT-LAMP 20 min and 25 min annealing times. The RT-LAMP reaction shows brighter bands for 25 min, but 20 min was chosen as the optimal time for swab sample amplification. (**B**) The result for RT-LAMP was read out on the LFA using biotinylated DNA probes (see Section 2.5). The LFA results were coherent with the results observed on the gel.

**Figure 5:**
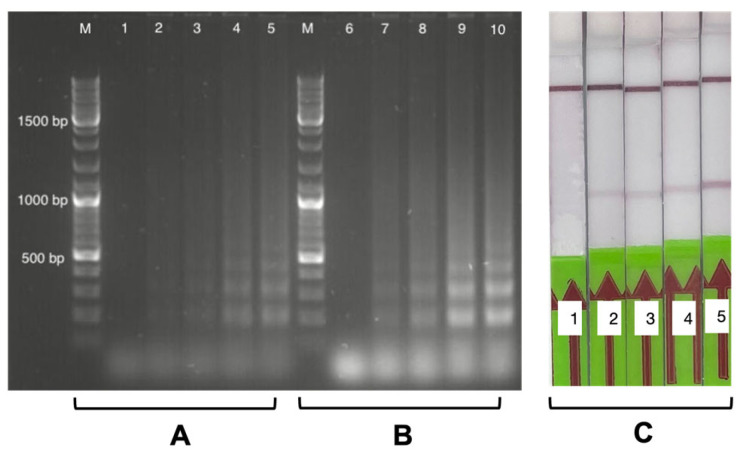
RT-LAMP for SARS-CoV-2 positive swab samples (CT value 18.55) in a 20 min of processing time. RT-LAMP results were read out on 1.5% agarose gel, and the gel image shows five RT-LAMP amplicon results before and after biotinylated-probe hybridization: one NTC and four quadruplets of positive sample with CT 18.55. (**A**) RT-LAMP results before biotinylated-probe hybridization: firstly, the M-100 bp DNA marker; then, NTC in lane 1; lanes 2 to 5 are 4 positive swab sample amplicons. (**B**) RT-LAMP results after biotinylated-probe hybridization: firstly, the M-100 bp DNA marker; lane 6 is NTC with all biotinylated-probes ABC; lane 7 is positive RT-LAMP amplicon with probe A; lane 8 is positive RT-LAMP amplicon with probe B; lane 9 is positive RT-LAMP amplicon with probe C; and lane 10 is positive RT-LAMP amplicon with all biotinylated-probes ABC. (**C**) LFA readout for the bi-labelled RT-LAMP amplicon: LFA 1 NTC with biotinylated-probes ABC; LFA 2 is positive RT-LAMP amplicon with probe A; LFA 3 is positive RT-LAMP amplicon with probe B; LFA 4 is positive RT-LAMP amplicon with probe C; and LFA 5 is positive RT-LAMP amplicon with all biotinylated-probes ABC.

**Figure 6:**
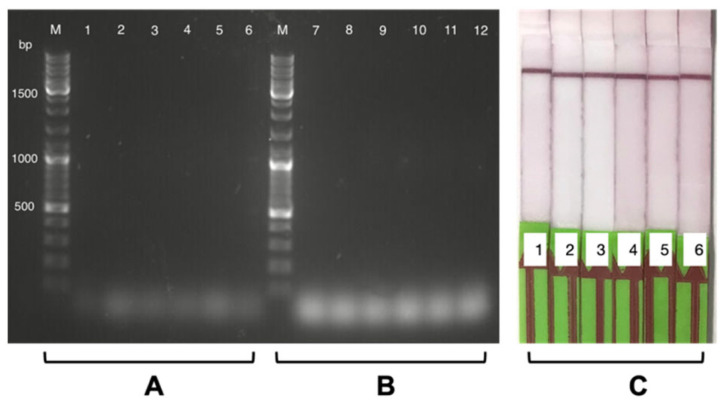
RT-LAMP for SARS-CoV-2 negative swab samples in 20 min of processing time. RT-LAMP results were read out on 1.5% agarose gel, and the gel image shows six RT-LAMP amplicon results before and after biotinylated-probe hybridization: two NTC and four negative samples. (**A**) RT-LAMP results before biotinylated-probe hybridization: firstly, the M-100 bp DNA marker; then, the NTC duplicate in lanes 1 and 2; lanes 3 to 6 are RT-LAMP amplicons of 4 different negative swab samples. (**B**) RT-LAMP results after biotinylated-probe hybridization: firstly, the M-100 bp DNA marker; then, the NTC duplicate with all biotinylated probes A, B, and C in lanes 7 and 8; lanes 9 to 12 are RT-LAMP amplicons of four different negative swab samples with all biotinylated probes A, B, and C. (**C**) LFA readout for the bi-labelled RT-LAMP amplicon: LFA 1 and 2 NTC duplicates with all biotinylated probes A, B, and C, and LFA 3 to 6 are RT-LAMP amplicons of 4 different negative swab samples with all biotinylated probes ABC.

LFA images were captured using an iPhone (in Figure 4B, Figure 5C and Figure 6C) and an IVD device purchased from MicroDiscovery GmbH (Berlin, Germany) for relative intensity readouts. The device has two main parts: (a) the custom LFA insertion socket connected to the spectrophotometer and (b) the smartphone, which has an in-built app for reading out the relative intensity of the test band and control band. However, the software provides all three values: T-line intensity, C-line intensity, and the T/C ratio.

### 2.9. Analytical Efficacy Calculations

Analytical efficacy for RT-LAMP-LFA results was evaluated using the following formula: Sensitivity = (number of true positives)**/**(number of true positives + number of false negatives), and specificity = (number of true negatives)**/**(number of true negatives + number of false positives). The results were also checked with the MedCalc statistical software. The McNemar statistical calculations were also confirmed with the MedCal statistical software 22.016.

## 3. Results and Discussion

### 3.1. RT-LAMP for SARS-CoV-2 Positive and Negative Throat Swab Samples as Template Optimisation

In total, 54 positive and 51 negative SARS-CoV-2 throat swab samples (see Section 2.1), clinically confirmed with the qRT-PCR quantified range of Ct values 18 to 34, were validated with RT-LAMP-LFA in this study. The RT-LAMP reaction mixture was prepared according to Table A2 (see Appendix A). The enzyme cocktail of Bst 3.0 polymerase, helicase, and reverse transcriptase was adapted from [27]. The RT-LAMP reaction mixture was subjected to isothermal amplification at 65 °C for 20 min, followed by inactivation at 83 °C for 2 min, and finally cooled to 4 °C for 2 min (see Section 2.3 and Table A3). For a qRT-PCR reaction, the heat inactivation of the whole swab sample is typically performed before the RNA extraction (from ≤200 Ll of the swab) [28,30]. In this study, the swab sample was collected from the throat (oropharyngeal) by trained personnel and immersed in ~2 mL of the Yocon-VTM. We tested 1 µL, 2 µL, 3 µL, 3.5 µL, and 4 µL volumes of crude throat swab samples (i.e., directly from the sample eluted in the Yocon-VTM) as templates in the RT-LAMP reaction mixture to maintain the rapidity of RT-LAMP within 20 min processing time. Additionally, 4 µL of the untreated swab sample was optimised for further RT-LAMP reactions. The crude swab sample composition (see Section 2.1) necessitated using a higher sample volume as a template in the RT-LAMP reaction compared to the generally used 1–2 µL of purified template for qRT-PCR or LAMP.

Most nucleic acid amplification techniques, such as qRT-PCR and isothermal methods, carry out amplification using cleaned and concentrated nucleic acid as a template, which incurs extensive pre-processing costs and time.

#### Optimisation of Processing Times for Swab Sample Amplification with RT-LAMP

We examined RT-LAMP with swab samples for 20 and 25 min using positive samples with different Ct values (see Figure 4A). All RT-LAMP products were run on a 1.5% agarose gel, and the characteristic concatemeric (ladder-like) pattern was observed, as shown in Figure 4A. There was a visible increase in the concatemeric signal intensity as the Ct value decreased, corresponding to the higher viral load in the sample. The gel electrophoresis result showed that RT-LAMP could amplify swab samples with the highest (33) and lowest (18.55) Ct values within 20 min of processing time at 65 °C.

### 3.2. Bi-Labelled RT-LAMP-Amplified Product for an LFA Readout

For the LFA readout used in this study, FITC and biotin require incorporation into the amplified product. As shown in previous studies, biotin-labelled dUTPs could be used to incorporate biotin into the amplified product [19,27]. In this study, the FITC-labelled LF-primer was incorporated during RT-LAMP amplification with an optimised RT-LAMP reaction mixture for swab samples (see Section 2.2), and an alternative approach with biotin-labelled DNA probes has been applied for biotin incorporation into the RT-LAMP-FITC amplicon (see Section 2.5). A non-template control (NTC) was run alongside all experiments to maintain and control the non-specificity of the reaction (if any). NTC also indicates the processing time limit for primer–dimer amplification. We observed that a 20 min processing time was optimal for the Ct value range of 18 to 33, as we could coherently read out the corresponding gel results on the LFA (see Figure 4B).

#### 3.2.1. Hybridization of Biotin-Labelled Probes with FITC-Labelled RT-LAMP Products

SARS-CoV-2 positive and negative samples were subjected to RT-LAMP (method in Section 2.1 and Section 2.2), wherein FITC-LF primer was incorporated during the reaction. After RT-LAMP amplification with FITC incorporation, the amplicon was heated to 52 °C for 2 min. Furthermore, the 0.25 µM end concentration of each biotin-labelled probe was introduced to the FITC-amplicon: Probes A, B, and C (see Appendix A Table A5). The mixture was left to incubate at room temperature for 3 min to ensure the specific hybridization of biotinylated probes. The gradual cooling at room temperature supports the hybridization process.

In other studies, a variety of immunochromatographic platforms have been discussed, which incorporate biotin and FITC/FAM labels during RT-LAMP/LAMP amplification, making it a “one-pot LAMP reaction” [12,20,21,22,27]. Though the reported assays are time-efficient (10 min for cDNA and 15 min for RNA) and closer to the POC setup, the sensitivity and specificity of these assays remained comparatively lower than qRT-PCR or lacked a standard clinical sample size evaluation (see overview in Table 1).

#### 3.2.2. Individual Specificity Test for Each Probe

Each biotinylated probe, i.e., A, B, and C (see Appendix A Table A4), was hybridized individually and verified for their binding affinities by reading the results of LFA (test band intensity). Each biotinylated probe produced a positive signal on the LFA with the RT-LAMP amplicon of a SARS-CoV-2 positive swab sample with a CT value of 18.55 (see Figure 5C). However, a combination of all three probes produced a higher-intensity test band on the LFA than using each probe individually (see Figure 5C). When only one biotinylated probe hybridizes with the RT-LAMP-FITC-amplicon, it only utilises one of the three designed regions available for biotin incorporated in each amplicon. On the other hand, when all three biotinylated probes hybridize, the incorporated biotin increases three times for each LAMP amplicon (see the abstract figure). The increase in localised incorporated biotin leads to principally better biotin capture by streptavidin on the LFA T-line and less hindrance between the two labels (FITC and biotin). This also supports and improves the anti-FITC-Ab capture of FITC molecules in the amplicon, generating a stronger-intensity T-line due to better AuNP aggregation. This result explains that the combination of all probes is more efficient than a single biotin probe to ensure the full potential of the RT-LAMP amplicon for generating an adequate signal on the LFA.

The strategic incorporation of biotinylated DNA probes shows a visible enhancement in the LFA test band intensity readout (compared to our previous work [26]) using a larger sample cohort. The biotinylated DNA probes used in our experiments provide a twofold advantage for improving the LFA assays. Firstly, the probes can be introduced through simple hybridization, carrying the second label needed for the LFA platform at relatively low temperatures (40–50 °C). Secondly, the probes improve the specificity of the assay since they introduce further gene-sequence information and that of the primers into the analysis.

#### 3.2.3. Positive and Negative Swab Sample RT-LAMP-LFA Readouts

A 1.5% agarose gel was run for reading out the RT-LAMP results: (a) RT-LAMP with an FITC-incorporated amplicon and (b) biotinylated-probe hybridization with an RT-LAMP-FITC amplicon. For SARS-CoV-2 positive swabs, we observed a characteristic concatemeric amplification pattern in the former case of RT-LAMP-FITC (see Figure 5A). For the latter case, an expected increase in the concatemeric pattern intensity was observed, proving that the probe binding enhanced the LAMP signal (see Figure 5B). No amplification (or non-specificity) was observed for the SARS-CoV-2 negative swab samples (see Figure 6A,B). To confirm the bi-labelling of the RT-LAMP amplicon, LFA was read out for each biotin probe hybridization experiment. We observed a positive test band appearing on the LFA for the positive swab samples in the CT range of 18 to 33 (see Figure 5C). Negative samples produced no test band signal on the LFA even after adding all three probes (see Figure 6C).

We have effectively demonstrated that crude swab samples in uVTM (54 positive and 51 negative samples) can be used “as is” as a template for RT-LAMP without any need for sample pre-treatment or the addition of nucleic acid concentrating filters, lysis solutions, or resins. The RT-LAMP result was visible in 20 min of RT-LAMP processing time for a CT value range of 18 to 33, with a Ct LoD of 33.

### 3.3. Analytical Efficacy

The RT-LAMP-LFA was performed for 54 positive and 51 negative SARS-CoV-2 swab samples. The RT-LAMP-LFA using the biotinylated-probe assay had a sensitivity of 98.11% and a specificity of 96.15%, with an accuracy of 97.14% and a McNemar (two-sided) *p*-value of <0.0001 with a CI of 95% (−4.18% to 2.28%). The RT-LAMP-LFA setup could detect the highest Ct value of 33, which corresponds to three RNA copies/µL (in reference to the RKI INSTAND standard) [26]. Though RT-LAMP has a complex fundamental principle, it seems on par with qRT-PCR with improved robustness, analytical sensitivity, and ease of diagnostic processivity.

### 3.4. Digitalization of the LFA Test Band Intensity Readout Using a Smartphone-Based IVD Device

**Figure 7:**
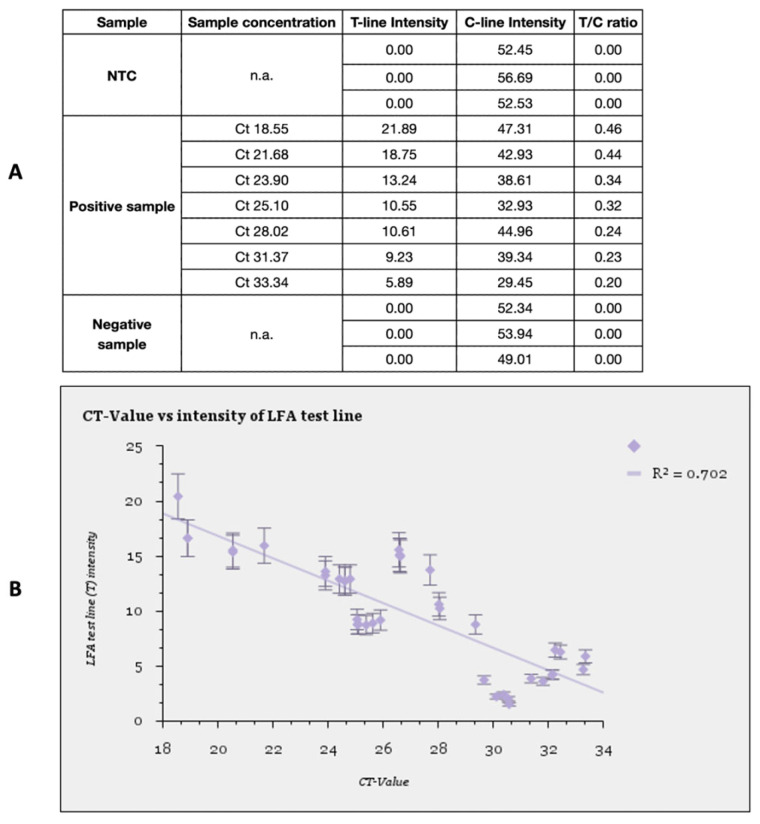
(**A**) The spectroscopic data of the LFA readout for NTCs, positive samples, and negative samples from the IVD device have been tabulated. The T-line intensities have been used to plot the graph in subfigure B. (**B**) Negative correlation curve between the Ct value of positive swab samples (*x*-axis) and the LFA test line intensity (*y*-axis) obtained after the readout of the bi-labelled amplicon. The regression coefficient is R^2^ = 0.702.

A smartphone-based in vitro diagnostic (IVD) device, which measures the relative intensity of the test band and control band on the LFA, was customised for our LFA setup (see Section 2.6). The relative intensity readout was recorded for 44 LFA results within the Ct value range of 18 to 33 (see Figure 7A). The test band intensity was visually observed to relate to the Ct value. Characteristically, the higher the Ct value of the SARS-CoV-2 positive swab sample, the lower the viral load in the sample and the lower the LFA test line intensity. Therefore, the T-line intensity was also inversely proportional to the Ct values obtained through qRT-PCR. The visual hypothesis was confirmed when a linear (negative) correlation with the regression coefficient R^2^ = 0.702 (see Figure 7B) was observed. Such correlations between test band intensities and Ct values aim towards a semi-quantitative result readout using the RT-LAMP-LFA technique.

A semi-quantitative approach with the IVD digitalization setup could help in improving contact tracing and surveillance, with better on-site applicability than PCR-based diagnostics. An LFA readout is a rather simple and versatile technique that can be adapted and digitalized for the detection of various pathogens [26,27,31]. Combining the RT-LAMP for nucleic acid amplification with LFA has been semi-quantified with a clear trend. The plot suggests the hypothesized correlation is possible but can be improved by using a larger sample cohort and reducing handling errors. Such a correlation plot for a specific test strip and assay setup can be standardized to resemble approved standardizations for qRT-PCR Ct values.

However, we want to stress that the meaning of this semi-quantification is limited due to the odds of sampling using an oropharyngeal swab, which is highly dependent on the personal behaviour of the sampling person. Hence, the following steps were taken: swab sample collection and dilution of collected swabs with a VT medium to influence the outcome of quantifying viral load. These factors significantly hinder the absolute quantification of the viral load of the patient on-site. Therefore, the RT-LAMP-LFA as a self-test would be deemed reliable only if performed repeatedly, eventually assessing this POCT setup for surveillance-based diagnostics.

## 4. Conclusions

During the COVID-19 pandemic, the pressure on qRT-PCR as a fundamental diagnostic method led to a clear understanding of various hurdles experienced in the field of diagnostics, e.g., the need for recruiting skilled personnel for sample collection and sample pre-treatment, additional time spent in sample transport to official centralized laboratories, lack of monitoring of infection for first-line workers and emerging new cases, inflated cost of medical supplies, surge and interference in production quality for PCR reagents, and more. The topic of reagent storage and durability is not described in this study, as it is difficult to analyse given a variety of environmental factors. On the other hand, regarding component temperatures and states, the enzyme *Bst* 3.0 loses its enzymatic activity when stored in the liquid state at > 4 °C temperatures. All other components, like DNA primers, salt, buffer, etc., can be stored at room temperature in a liquid or dry state (lyophilised). Hence, looking for a modified *Bst* enzyme that retains the activity after dissolving from the lyophilized state is the next important step.

In this study, we demonstrate a technological design of an RT-LAMP-LFA-based biosensor, exclusively using crude swab samples in uVTM “as is” as a template. Overall, the RT-LAMP-LFA assay took 35 min: 25 min RT-LAMP (at 65 °C) + 3 min biotinylated probe annealing (at <60 °C) + 3 min incubation for hybridization (at room temperature) + 2 min LFA readout + 2 min LFA analysis using a smartphone. The efficacy of the complete assay (54 positive and 51 negative samples) shows a sensitivity of 98.11% and a specificity of 96.15%, with an accuracy of 97.14% and a McNemar (two-sided) *p*-value of <0.0001 with a CI of 95% (−4.18% to 2.28%). In conclusion, the crude swab sample reduces sample pre-treatment efforts, and the semi-quantification corresponds with the LFA results, demonstrating the feasibility of a molecular point-of-care diagnostic platform for an ASSURED (affordable, sensitive, specific, user-friendly, rapid and robust, equipment-free, and deliverable) on-site applicable biosensor, following the WHO guidelines [9,20]. A semi-quantified RT-LAMP-LFA POC technique with the described approach could boost cheaper and enhanced surveillance, monitoring, and contact tracing of infections in a pandemic, epidemic, endemic, etc. In the future, DNA probes could be used as a recognition element, directly immobilised on the LFA itself, paving the way for a paper-based DNA array. This approach opens the possibility of label-free multiplexing in the LFA format, thus broadening the scope of LFA applications in POCT situations. This approach may be an alternative to the recently proposed microfluidic multiplexing methods [32,33,34]. This setup attempts to bridge the gap between expensive qRT-PCR diagnostics and the efficiency of rapid antigen tests.

## Figures and Tables

**Table 1:** A brief overview of the versatile use case of isothermal amplification with RT-LAMP (different parameters) and mostly LFA (different LFA chemistries and principles) as a readout platform, including this study’s results. In these cases, the templates used for RT-LAMP are extracted viral RNA from swabs using one of two nucleic acid extraction techniques. In these cases, the processing time of RT-LAMP is in the range of 15 min minimum to 60 min maximum at 65 °C. This overview of RT-LAMP-LFA depicts one of the main limitations, i.e., nucleic acid extraction from the initial swab sample collected.

Organism	Template	Sample Size	Amplification Technique	Processing Time	Readout	Efficacy	References
SARS-CoV-2 E-gene	Extracted RNA	55 positive 55 negative	RT-LAMP	30 min (63 °C)	Fluorometric detection	97.2% sensitivity 100% specificity	[9]
SARS-CoV-2	Extracted RNA	25 positive 25 negative	RT-HDA	30 to 90 min (42 °C)	LFA	100% sensitivity 100% specificity	[12]
SARS-CoV-2 *E-* and *N*-gene	Extracted RNA	25 positive 25 negative	RT-LAMP	60 min (65 °C)	LFA	100% sensitivity 83.3% specificity	[12]
SARS-CoV-2 *N*-gene	Extracted RNA	150 positive 110 negative	RT-LAMP	1 h (65 °C)	pH based colorimetric	92.7% sensitivity 93.6% specificity	[18]
SARS-CoV-2 *N* and ORF1ab gene	Lysed RNA	12 synthetic positive samples, 8 positive swab samples, 4 negative swab samples from healthy individuals, 6 negative samples (Mycoplasma infection)	RT-LAMP	30 min (60–65 °C)	LFA	100% sensitivity 100% specificity	[22]
SARS-CoV-2 *N*-gene	Extracted RNA and cDNA	82 positive and non-template control	RT-LAMP	15 min (65 °C)	LFA	95.4% sensitivity 96.5% specificity	[27]
SARS-CoV-2 *N*-gene	Swab sample in uVTM	54 positive and 51 negative SARS-CoV-2 throat swab samples	RT-LAMP	20 min (65 °C)	LFA	98.11% sensitivity 96.15% specificity	This study

## Data Availability

Data are contained within the article.

## Appendix A

**Table A1:** LAMP primers and PCR primers for *N*-gene 466 bp amplification were designed using Primer Explorer.

Primers	Sequences (5′ -> 3′)	Length (nt)
F3	CCCAAAATCAGCGAAATGCA	20
B3	AGCCAATTTGGTCATCTGGA	20
FIP	TGTTTTGATCGCGCCCCACTGATTACGTTTGGTGGACCCTC	41
BIP	TGCGTCTTGGTTCACCGCTCATTGGAACGCCTTGTCCTC	39
FITC-LF	**FITC—**TCCATTCTGGTTACTGCCAGTTGAA	25
LB	ACTCAACATGGCAAGGAAGACCTTA	25
Fwd_PCR	GACCCCAAAATCAGCGAAAT	20
Rev_PCR	TGTAGCACGATTGCAGCATTG	20

**Table A2:** RT-LAMP reaction list of reagents for 25 µL reaction volume.

Reagent	Stock Concentration	End Concentration	Company
10X Isothermal buffer	10 X	1 X	New England BioLabs Inc.
MgSO_4_	100 mM	6 mM	
ATP	10 mM	0.4 mM	
dNTP mix	40 mM	5 mM	
F3 Primer	10 µM	0.6 µM	Biomers.net GmbH
B3 Primer	10 µM	1 µM	
FIP Primer	10 µM	0.6 µM	
BIP Primer	10 µM	0.6 µM	
FITC-LF Primer	20 µM	1 µM	
LB Primer	10 µM	0.6 µM	
Tte UvrD Helicase	20 µg/ml	0.8 µg/ml	New England BioLabs Inc.
WarmStart^®^ RTx Reverse Transcriptase	15 U/µL	0.6 U/µL	
*Bst* 3.0 DNA polymerase	8 U/µL	0.32 U/µL	
Nuclease free PCR grade water	—	—	Roth Industries Inc.
Swab Sample	CT 18 to CT 32		In.vent Diagnostics GmbH

**Table A3:** RT-LAMP reaction temperature cycling.

Lid Temperature 72 °C		
**Step**	**Temperature (°C)**	**Duration (min)**
**Annealing**	65	20
**Inactivation**	83	2
**Cooling**	4	2

**Table A4:** DNA probes designed for *N*-gene of SARS-CoV-2 were biotinylated at the 5′ end.

Probe	Sequences (5′ → 3′)	Tm (°C)	Length (nt)	GC %
**A**	**Biotin**-TAAACCTTGGGGC	40	13	53.8
**B**	**Biotin**-ACCAAGACGCAG	38	12	58.3
**C**	**Biotin**-GAGAGCGGTGA	36	11	63.6

**Table A5:** Steps for biotinylated DNA probes hybridization to FITC-labelled RT-LAMP amplicon.

Step		Temperature (°C)	Duration (min)
1. Denaturation of amplified products		52	2
2. Biotin probe (0.25 μM)	Probe A	46	2
	Probe B	39	2
	Probe C	39	2
	All 3 probes	46	2
3. Cooling		RT	5
